# Empowering people for sustainable development: the Ottawa Charter and beyond

**DOI:** 10.7189/jogh.07.010308

**Published:** 2017-06

**Authors:** Sagar Dugani, Zulfiqar A. Bhutta, Niranjan Kissoon

**Affiliations:** 1 Department of Medicine, University of Toronto, Toronto, Ontario, Canada; 2 Centre for Global Child Health, The Hospital for Sick Children, Toronto, Ontario, Canada; 3 British Columbia Children’s Hospital and University of British Columbia and Vancouver, British Columbia, Canada

**Figure:**
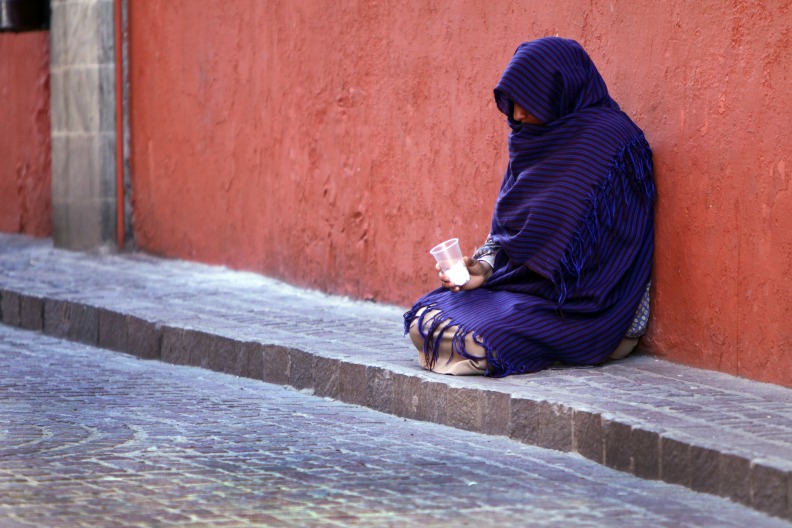
Photo: Women beggar, by Tomas Castelazo, own work, CC BY-SA 3.0

In November 1986, 212 participants from 38 countries convened in Ottawa, Canada, at the first International Conference on Health Promotion organized by the World Health Organization (WHO), Health and Welfare Canada, and the Canadian Public Health Association. Although not representative of the entire globe, these participants adopted the *Ottawa Charter* on health promotion as “*the process of enabling people to increase control over, and to improve, their health*”. The five action areas identified were to: build healthy public policy; create supportive environments; strengthen community action; develop personal skills; and re-orient health care services, toward prevention of illness and promotion of health [1–3]. The *Charter* laid the foundation for subsequent efforts in advancing global health over the next 30 years, including development of the Millennium Development Goals (MDGs). The MDGs have given way to a more robust and comprehensive agenda, the Sustainable Development Goals (SDGs) [4], underpinned by the principles of reducing inequities and universal health coverage (UHC).Thus the vision of the *Charter* is foundational to progress in health care over the last 30 years. Here, we review the progress made and discuss the unfinished agenda of the *Charter* as well as opportunities to accelerate its objectives in the context of UHC and the SDGs, through collaborations of international and local health organizations.

The notion of UHC was articulated earlier in September 1978 when the *Alma Ata Declaration* on primary health care was adopted at a joint conference of the WHO and United Nations Children’s Emergency Fund (UNICEF) [5]. This declaration emphasized the importance of ‘health for all’, a message strengthened through the *Charter* and in the WHO strategy to ensure ‘*Health for All by 2000*’. In this regard, the *Alma Ata Declaration* and the *Charter* were seminal documents that shaped the global health agenda. The *Charter* urged nations to advocate, enable, and mediate, in order to achieve progress in the five action areas [3]. While the ambitious agenda of the *Charter* and *Health for All* were not fully realized, this heralded the era of the MDGs that guided the global health agenda from 2000–2015, and yielded substantial but uneven gains. The MDGs have been replaced by the SDGs, which comprise of 169 targets, and were adopted in September 2015 by 193 nations [6]. The Ottawa Charter notes that “*peace, shelter, education, food, income, a stable eco–system, sustainable resources, social justice, and equity*” are fundamental resources for health [3], and the SDGs address these domains. Countries have achieved different levels of success in these domains, and rather than prioritize the relative importance of domains for a particular country, we propose that countries acknowledge the interdependence of these different domains and that health cannot be improved in isolation of these domains. While the ambitious SDG agenda reflects the complexity and intersectoral relations required to achieve optimal health, it has also established broad, incompletely defined targets that are difficult to quantify, measure, implement, and evaluate. In addition, achieving UHC to ensure people have access to affordable, high–quality health care is a core element and enabler of success of the SDGs. However, achieving UHC and thus the SDGs will remain beyond the reach of many nations that are struggling with geopolitical and economic instability. Given the known “inverse equity hypothesis” of inequities worsening as new interventions are introduced [7], the implementation of UHC and new interventions for SDG3 must be undertaken with a specific focus on reaching all and reducing inequities.

Empowering of individuals and communities to advocate for and improve access to health care is central to success of the *Charter* and all subsequent global health frameworks. However, empowerment of the disadvantaged is difficult and three decades after the adoption of the *Charter*, a key unanswered question is *how best to empower people.* Empowering and enabling people to take charge of and improve their health are weighty undertakings. Within the health care framework, it requires health literacy and access to health information; access to affordable health services and medicines; and, a resilient patient–centered health care system. More importantly, health care cannot be realized without exploring the impact of non–health sectors including geopolitical, economic, and financial stability; policies on manufacturing and pricing of medicines; and political will to implement and evaluate change. In many parts of the world achieving equity in health outcomes based on socioeconomic gradients, gender or geography requires policies and empowered communities to exercise their political capital in achieving these goals. In addition, global armed conflicts result in reduced access to health services, forced migration, social breakdown, and heightened stress, all of which have negative consequences on health [8]. The Rio 20+ summit aimed to advance sustainable development, and highlighted the impact of environmental changes (for example: climate change, loss of biodiversity, using indoor cooking fuels) on health [9]. It is becoming more apparent that environmental pollutants can have a significant impact on overall health [10,11].

*How do we empower people?* As outlined in the *Charter* and emphasized by the SDGs, the current focus is on enabling people to take control of their health. To achieve this, interventions at the community and individual levels are required. Mass public health campaigns can educate communities on behaviors including use of alcohol, tobacco, and safer–sex practices [12]. The value of community engagement and support groups has been well documented through a range of large scale evaluations in maternal and newborn health [13]. At the individual level, people should have access to affordable, high–quality health care, which is at the core of achieving UHC. However, access is problematic and is likely to assume crisis proportions as the WHO projects a shortage of 12.9 million health workers over the next two decades [14]. Moreover, low and middle–income countries (LMICs) will be severely affected by the ongoing migration of their workforce to high–income countries (HIC). In addition, the unaffordability of essential medicines threatens to increase the global burden of cardiovascular, respiratory, and other chronic, non–communicable illnesses. Finally, all nations will require evaluation of local and regional health outcomes to identify additional gaps, develop interventions, and monitor impact on health outcomes. Without clarity on *who*, *how*, and *how often* health outcomes should be monitored, and without effective monitoring systems in place, nations will be unable to improve outcomes and achieve many of the 169 targets of the SDG. Therefore, successful empowerment of people will rely on an educated population that is aware of these ground realities and can advocate for change. The Treatment Action Campaign (TAC), formed in 1998, has advocated for improved HIV care in South Africa has focused on empowering people by improving health education, increasing access to essential medicines, taking on government policy, and effecting social change [15]. The successes and challenges experienced by the TAC can help other global organizations in their efforts to empower people.

*Where do we go from here?* Thirty years after the *Charter*, we are still working toward the unfinished agenda of empowering people, and there remain several challenges. A study of semi–structured interviews with 22 health leaders from LMICs and HICs identified challenges associated with empowering people: first, the term ‘empowerment’ may be considering ‘soft’ or ‘opaque’ and does not easily translate into action; second, empowering people shifts the power dynamic from governments to citizens, and may not be viewed positively in all countries; and, people in many countries have been disenfranchised, and instilling confidence and hope remains challenging [16]. To achieve any of the aspirational SDGs, we will require a holistic effort from HICs to assist LMICs monitor their health systems and reach their health targets. Many countries are moving toward a more nationalistic agenda, and international efforts (particularly, to help LMICs) are likely going to decline. The Ebola crisis largely affected LMICs, and yet, it was argued that HICs are obligated to contribute to humanitarian assistance and global justice [7]. However, non–communicable diseases will require a longer–term commitment, and LMICs will rely on HICs for guidance and financial assistance in improving health. We will also require a concerted approach to reduce the cost of medicines and increase access to affordable health care. In many instances, lack of political support has been a major obstacle to implementing the health agenda. These cannot be achieved in the absence of reducing road injuries, improving fuel sources to reduce air pollution, reducing wars and conflict, improving political and environmental stability, managing population growth, and ultimately, sharing experiences with other nations that may be at a different point on the trajectory toward achieving UHC and the SDGs. All of these facets are important, and nations will have to articulate their individual developmental agenda based on their local needs and priorities. The SDGs should serve as a guide and framework for ongoing improvement and robust implementation research to guide policy. An immediate priority therefore is to develop an agenda for implementation that engages and empowers people.
